# Validation of the Danish version of the Carer Experience Scale in family caregivers of people with dementia

**DOI:** 10.1002/alz70858_100790

**Published:** 2025-12-25

**Authors:** Emma Sofie Kjær Pedersen, Laila Øksnebjerg, Gunhild Waldemar, Janet Janbek, Ann Nielsen, T Rune Nielsen

**Affiliations:** ^1^ Danish Dementia Research Centre, Copenhagen University Hospital ‐ Rigshospitalet, Copenhagen, Denmark; ^2^ University of Copenhagen, Copenhagen, Denmark; ^3^ Department of Clinical Medicine, University of Copenhagen, Copenhagen, Denmark

## Abstract

**Background:**

Caring for a family member with dementia places significant strain on family caregivers, affecting their quality of life. When evaluating the economic effects of psychosocial interventions, it is essential to apply outcome measures that reflect family caregivers’ everyday experiences. However, finding such validated measures can be challenging. The Carer Experience Scale (CES) aims to capture aspects of quality of life beyond health. We aimed to validate a Danish version of the CES by assessing its discriminative and convergent validity in family caregivers of people with dementia and establishing its scale‐ and test‐retest reliability.

**Method:**

375 family caregivers of people with dementia participating in the Danish DemTool intervention trial completed a baseline questionnaire, including six quality‐of‐life and wellbeing measures. Discriminant validity was assessed by comparing different caregiver situations and levels of caregiver strain. Convergent validity was assessed by correlating the total CES score and CES domain scores with the Neuropsychiatric Inventory Caregiver Distress Scale (NPI‐D), European Quality of life – 5 Dimensions – 5 Levels instrument (EQ‐5D‐5L), European Quality of life visual analog scale (EQ‐VAS), the World Health Organization Wellbeing Index (WHO‐5), and the University of California, Los Angeles Three‐Item Loneliness Scale (UCLA 3‐item). Scale reliability was measured with Cronbach's alpha, and test‐retest reliability was determined using the Intraclass Correlation Coefficient (ICC) at two different time points.

**Results:**

The analysis of discriminative validity indicated that the CES effectively distinguishes between different levels of caregiver strain. Further, it showed that CES was not affected by the sex of caregivers, education, age, civil status, and the care‐receiver sex, age, and residence. The convergent validity analysis showed moderate correlations of the CES with NPI‐D, WHO‐5, and UCLA 3‐item, and weak correlations with EQ‐5D‐5L and EQ‐VAS. The analysis of the reliability of the CES showed a low internal consistency (α=0.53) and a high test‐retest reliability (ICC=0.77).

**Conclusion:**

The Danish CES showed acceptable psychometric properties, and this study provides further evidence for the CES in family caregivers of people with dementia. CES provides an everyday life approach that may more effectively assess the impact of family caregiver interventions.

